# Estimating global public health security preparedness capacity: The contribution of SPAR and JEE

**DOI:** 10.1016/j.dialog.2026.100282

**Published:** 2026-01-17

**Authors:** Kamal Bishowkarma, Cynthia Bell, Shanlong Ding, Robert Nguni, Luca Vernaccini, Peter Mala, Stéphane de la Rocque, Jun Xing, Dick Chamla, Mary Stephen, Reuben Samuel, Ihor Perehinets, Phuong Nam Nguyen, Nirmal Kandel, Stella Chungong

**Affiliations:** a World Health Organization, Headquarters, Geneva, Switzerland; b WHO Regional Office for the Western Pacific, Manila, Philippines; c WHO Regional Office for Africa, Brazzaville, Republic of Congo; d WHO Regional Office for the Eastern Mediterranean, Cairo, Egypt; e WHO Regional Office for South-East Asia, New Delhi, India; f WHO Regional Office for Europe, Copenhagen, Denmark

**Keywords:** Global health security, International health regulations (IHR) (2005), State parties annual self-assessment (SPAR), Joint external evaluation (JEE), Capacity assessment, Pandemic preparedness

## Abstract

**Background:**

To promote transparency and mutual accountability in global public health security among WHO States Parties, Article 54 of the International Health Regulations (2005) (IHR) obliges State Parties to regularly report capacities to prevent, protect against, control, and provide a public health response to the international spread of disease. Two prominent tools for assessing capacities, a mandatory State Parties Annual Self-Assessment (SPAR) and a voluntary Joint External Evaluation (JEE), cover similar concepts and structure, but agreement between the tools has not been assessed in a long term global analysis.

**Methods:**

This ecological study compared quantitative capacity scores from 1445 indicator-matched paired observations from 108 SPAR and JEE assessments completed in the same year, by 93 States Parties, between 2016 and 2023. Mixed effects methods were used to estimate mean agreement for each indicator, comparing tool editions/years, regions, and income groups.

**Findings:**

Overall, SPAR scores were higher than JEE scores, with the least agreement observed with indicators scoring near Level 3–4 capacity. However, consistency between SPAR and JEE evaluations improved recently, particularly in the latest 2022–2023 editions where 25 of the 28 matched indicator capacity scores were not significantly different on average. Three indicators with significant score disagreement pertained to infection prevention and control, health-care association infection surveillance, and national IHR focal point functions.

**Interpretation:**

Improved alignment between SPAR and JEE, particularly in recent editions, combined with the identification of remaining indicator disagreement, strengthens the evidence base for continued improvement in these essential assessment tools.

## 1 Research in context

Discussion on how countries best assess and report capacities for global public health security has intensified, particularly with lessons from COVID-19 and the need to improve preparedness to better mitigate future pandemics. The WHO introduced the IHR State Parties Annual Self-Assessment (SPAR) tool to help countries fulfill Article 54 of the International Health Regulations (IHR) (2005) reporting obligations for transparency and accountability. Recognizing potential self-reporting bias, the Joint External Evaluation (JEE) was introduced later as a complementary instrument to SPAR, both being integral components to the IHR Monitoring and Evaluation Framework (IHRMEF). Regional experience and prior studies noted SPAR scores were typically higher than JEE. However, these studies were limited by number of countries included, time period examined, and tool editions used. This study confirms higher SPAR scores in earlier editions but expands the analysis period, showing improved conceptual indicator alignment and score consistency between the tools in the most recent 2022–2023 editions, with 28 matched indicators and a reduced mean difference of 6 points from 20 points in initial editions. A detailed analysis of each matched indicator identifies areas of greatest and least agreement. The quantitative findings offer insights into how best to interpret scores from the self-assessments and external evaluations. Most importantly, these findings emphasize the importance of tool updates and adaptations, ensuring accurate description, clear definitions, and consistent measurement of capacities needed to prevent, protect against, control, and provide a public health response to the international spread of disease.

## 2 Introduction

The International Health Regulations (IHR) (2005) is a legally binding instrument of international law that creates rights and obligations for countries to prevent, protect against, control, and provide a public health response to the international spread of disease. [1] Annual reporting obligations, introduced in Article 54 of the IHR (2005) and emphasized in World Health Assembly (WHA) resolutions WHA61.2 and WHA71.15, was a pivotal step toward ensuring transparency and mutual accountability among WHO States Parties in strengthening global public health security. [2], [3] Ensuring reliability of the reporting tools is instrumental not only for IHR compliance but also for achieving and measuring progress toward the WHO Fourteenth General Programme of Work (GPW14) strategic objective to prevent, mitigate, and prepare for health risks from all hazards. [4]

In alignment with these principles, the WHO developed the IHR Monitoring Questionnaire in 2010, to facilitate the systematic self-assessment and reporting process. The IHR Monitoring Questionnaire served as the primary instrument for States Parties to annually assess and report on their capacities to implement the IHR. Emphasizing mutual accountability between States Parties and the WHO, the tool was designed to be comprehensive and multidisciplinary, incorporating inputs from all government sectors relevant to national IHR capabilities. [2], [3] The tool was revised and enhanced in subsequent editions, with the 1st edition of the IHR States Parties Self-Assessment Annual Reporting (SPAR) tool released in 2018 that covered 13 capacities with 24 indicators and then 2nd edition released in 2021 covering 15 capacities with 35 indicators which integrated lessons learned from the COVID-19 pandemic. These SPAR revisions and the rollout of the e-SPAR online reporting platform reflect adaptability to the changing global health landscape and modern approach to monitoring and evaluating capacities.

In response to evolving global public health security needs following lessons from the 2014–2016 West African Ebola outbreak, the WHO introduced the Joint External Evaluation (JEE) tool in February 2016 based on recommendations from the IHR Review Committee to “move from exclusive self-assessments to approaches that combine self-evaluation, peer review and external evaluations involving a combination of domestic and independent experts”. [5], [6], [7] JEEs incorporate voluntary country participation, a multisectoral approach by both external teams and host countries, transparency and information sharing, and the public release of reports. Since 2016, three editions of the JEE have been published: JEE 1st edition (2016), JEE 2nd edition (2018), and JEE 3rd edition (2022). The JEE 2nd edition marked significant improvements, including the introduction of new financial indicators and restructuring of technical areas to ensure more granular insights into national public health security mechanisms with key findings presented as priority actions for each of the 19 technical areas. [6], [7] A pivotal revision came with the JEE 3rd edition, developed post extensive consultations in 2021 in response to the COVID-19 pandemic when studies were unable to show a clear association of SPAR or JEE scores with COVID-19 outcomes. [8], [9], [10] This latest JEE edition, which includes 19 technical areas, underscores the importance of health equity, with added provisions to identify support for vulnerable populations and foster a more inclusive, multisectoral One Health approach to public health security preparedness. [7]

Given their complementary purpose in supporting States Parties in assessing and improving IHR core capacities for health emergencies, both SPAR and JEE are integral components of the IHR Monitoring and Evaluation Framework (IHRMEF). Many of the JEE technical areas and SPAR core capacity areas intersect, with indicators that address similar technical areas and measure the same concepts. Previous studies by Stone et al. in 2022, Razavi et.al in 2021, and Kandel et al. in 2018 have identified both direct and partially matched indicators between SPAR and JEE, underscoring their potential complementary properties. [11], [12], [13] Kandel et al. notably reported an improvement in agreement between SPAR and JEE following the revision of the JEE tool in 2018. This study's motivation stems from the need for comparable indicators across the IHRMEF, enhancing its utility for accurate reporting and strategic planning.

While the primary goal of these tools is to support IHR reporting, they are also used to inform broader decision-making, prioritize investments, and ascertain actual preparedness status for major health emergencies. However, debates exist on how to accurately define and measure countries' preparedness, and questions remain regarding the complementarity and agreement between the two tools across different editions over time. The concerns about the global communities' lack of preparedness for large-scale disease threats, initially prompted by the 2014–2016 West African Ebola outbreak and profoundly reinforced by the COVID-19 pandemic, highlighted an unexpected lack of preparedness even in countries reporting high preparedness levels [8], [9], [10]. For instance, the Global Health Security Index, which utilizes measures from the JEE, showed significant discrepancies with actual national responses in many high-scoring countries that struggled to control the pandemic. [14] Improving the reliability and consistency of these existing measures is essential to avoid confusion and misdirection in defining needed actions and investments, and to enhance their validity as indicators of true preparedness.

To address these challenges, this study longitudinally analyzes SPAR and JEE assessment data from 2016 to 2023, a longer period than examined in previous studies, to determine if these tools show improved alignment in indicator concepts and matched indicator scores. This analysis takes an important step to identify areas where the tools agree or disagree, which may indicate where concepts need refinement, indicators may need to be redefined, or new indicators may be required to cover a broader scope. Improving the indicator agreement of these complementary tools within the IHRMEF is essential to enhance their validity as indicators of true preparedness. Ultimately, this study will strengthen the evidence base for continuous improvement of these essential assessment tools, aiding results interpretation, and supporting countries in meeting their IHR obligations.

## 3 Methodology

### 3.1 Study design and data source

The analysis was an ecological study design that included all 196 WHO States Parties from all six WHO regions. The primary inclusion criterion for a State Party's data was the completion of both a SPAR and a JEE assessment in the same calendar year within the 2016–2023 period to allow a direct indicator-matched paired comparison. The data for indicator level scores were collected and consolidated for each State Party from the SPAR and the JEE platforms (eSPAR and SPH portals) from January 2016 to December 2023, inclusive. For simplicity, we used the general term “SPAR” when referring to the assessments that include the IHR Monitoring Questionnaire, SPAR 1st edition, and SPAR 2nd edition.

### 3.2 Data matching

There were four time periods with unique SPAR and JEE edition indicator matches (Table 1). While SPAR versions are introduced on an annual basis with the same version used for all States Parties for any given year, JEE edition use by States Parties can vary within a year depending on the month published, thus it was verified which JEE version for each country was used in years where a new JEE edition was published (years 2018 and 2022). To assess conceptual alignment, the relevant indicators from JEE and SPAR were considered as matched if they aligned word for word, or if they measured the same concept despite some differences in wording. A group of experts from the WHO headquarters matched the indicators across the three editions of SPAR and the three editions of JEE and between each SPAR and JEE edition pair (as shown in Table 1) to identify directly matched indicators (Annex Table 1–3).

**Table 1:** Years and editions of SPAR and JEE from 2016 to 2023.

	January 2016 to January 2018	February 2018 to December 2020	January 2021 to June 2022	July 2022 to December 2023
*Number of State Parties included based on JEE completion*	*n* = 52 JEEs	*n* = 31 JEEs	*n* = 3 JEEs	*n* = 22 JEEs
*SPAR Edition*	IHR Questionnaire	SPAR 1st edition	SPAR 2nd edition	SPAR 2nd edition
*JEE Edition*	JEE 1st edition	JEE 2nd edition	JEE 2nd edition	JEE 3rd edition

### 3.3 Data analysis

The primary outcome of the study was indicator-level difference between JEE and SPAR indicator scores available for the same year for each State Party. Both SPAR and JEE scores are reported as five level categories with level 1 the lowest performing and level 5 the highest performing. We multiplied the assigned level with the absolute number of 20 to align scores to the 0 to 100 scale range, as is noted in the SPAR guidance documents. [2], [3] If two indicators in a tool mapped to one indicator in the other tool, the arithmetic mean of the two indicator scores was used. To calculate the difference between indicator-matched SPAR and JEE scores, the study used the following equation.

$$\mathrm{Indicator}\;{\mathrm{matched difference}}_{\mathrm{ijk}}={\mathrm{SPAR score}}_{\mathrm{ijk}}-\mathrm{JEE}\;{\mathrm{score}}_{\mathrm{ijk}}$$

where i indicates the State Party, j indicates the year/edition, and k indicates matched indicator from SPAR and JEE. After calculation of each State Party indicator-matched difference, the mean and 95% confidence intervals (CI) were estimated using mixed effects models to account for repeated measures across States Parties and years with random effects for both States Parties and years nested within States Parties. All other factors were considered as fixed effects in the models. Differences were calculated at global, regional, and World Bank income group levels, as well as for each specific indicator within and across all years/editions. Bland-Altmann plots were used to determine limits of agreement and explore trends in difference across the magnitude of the capacity scores. All analyses were completed using R statistical software with packages lme4, emmeans, blandr, and tidyverse. [15], [16], [17], [18], [19]

## 4 Results

The final analysis included 1445 indicator-matched SPAR and JEE paired observations from 108 completed JEEs among 93 States Parties, with the majority of JEEs completed in the African region (56, 52%) and the fewest JEEs completed in the Americas region (2, 2%) [Table 2]. Not all indicators could be directly matched, with differences associated with the successive versions of the tools. In total, 29 indicators were included as direct matches between SPAR capacity and JEE technical concepts: 11 indicators in 2016–2017, nine indicators in 2018–2020, 10 indicators in 2021–2022, and 28 indicators in 2022–2023. There were nine indicators that matched across all editions from 2016 to 2023.

**Table 2:** Indicator-matched mean difference between SPAR and JEE scores from 2016 to 2023, by global, WHO regions, and World Bank income groups. The right column indicates number (%) of paired SPAR-JEE assessments from the earliest editions (2016–2017) to determine potential confounding between grouping and timing of assessments.

Level of Analysis		Mean difference [95% CI]	Number completed JEE and SPAR in same year	Number (%) completed in 2016–2017: JEE 1st Edition and IHR Questionnaire
Global		13.2 [10.3, 16.0]	108	52 (48%)
WHO regions	African Region	11.1 [7.1, 15.0]	56	24 (43%)
	Region of the Americas	13.3 [−6.8, 33.5]	2	1 (50%)
	Eastern Mediterranean Region	17.6 [10.3, 25.0]	15	10 (67%)
	European Region	8.0 [0.7, 15.3]	15	5 (33%)
	South-East Asia Region	22.9 [13.8, 32.0]	10	7 (70%)
	Western Pacific Region	15.8 [6.6, 25.0]	10	5 (50%)
World Bank income groups	Low income	11.5 [5.6, 17.4]	24	14 (58%)
	Lower-middle income	16.9 [12.1, 21.6]	34	19 (49%)
	Upper-middle income	15.0 [8.2, 21.8]	18	6 (33%)
	High income	11.0 [4.4, 17.6]	19	13 (68%)

The global mean difference between SPAR and JEE was 13.2 reflecting that SPAR was 13 points higher than JEE on average across all countries, years, and matched indicators [Table 2]. Regionally, the European region matched indicator scores were most comparable with mean difference of 8.0 from 15 completed SPARs and JEEs, while the South-East Asian region displayed the largest matched indicator difference with mean 22.9 among 10 completed SPARs and JEEs. Of note, the majority of European region JEEs were completed more recently (67% completed after 2017) while 70% of the South-East Asian regions' JEEs were completed using earlier tool editions in 2016–2017. The results showed more consistency across World Bank income group than WHO regions. On average, SPAR scores were 11 points higher than JEE in high-and low-income countries while 17 points higher in the lower-middle-income countries.

**Fig. 1:**
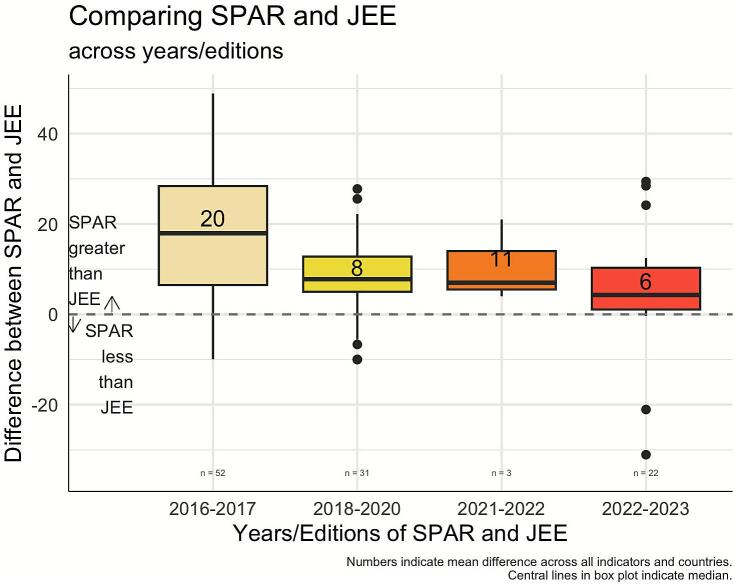
Indicator-matched mean difference between SPAR and JEE scores from 2016 to 2023, by years/editions.

The SPAR and JEE trends showed improved alignment across years and editions [Fig. 1, Annex Fig. 1]. In the first edition, 2016–2017, SPAR scores were 20 points higher than JEE on average. The alignment improved in the second edition, 2018–2020, with SPAR scores only 8 points higher than JEE on average. Moreover, the most recent editions of SPAR and JEE showed further improved alignment in 2022–2023 with SPAR only 6 points higher than JEE on average. While the 2021–2022 period had a slight increase in the gap, the COVID-19 pandemic restricted the completion to only three States Parties thus should be considered in the result interpretation. Improved SPAR-JEE agreement in the 2022–2023 editions is evident across most regions, with notable exceptions in the Americas region, where data was insufficient, and the European region, where agreement decreased in recent editions [Annex Fig. 2].

**Fig. 2:**
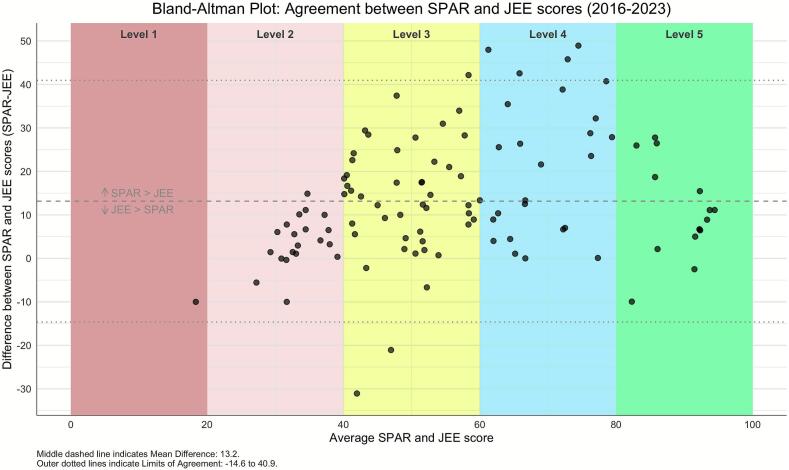
Distribution of indicator-matched mean and difference of SPAR and JEE scores from 2016 to 2023 demonstrates that the largest differences are seen at scores.

To further explore concordance between SPAR and JEE across all 108 paired assessments in 93 States Parties, we used Bland Altman plots to determine if agreement between SPAR and JEE scores is related to the level of capacity. As shown in Fig. 2, States Parties with levels 3 and 4 average capacity status (score between 40 and 80) across all matched indicators showed the least agreement between SPAR and JEE scores. Conversely, States Parties with lower capacity (score less than 40) and higher capacity (scores higher than 80) tended to have more agreement between SPAR and JEE matched-indicator scores.

**Fig. 3:**
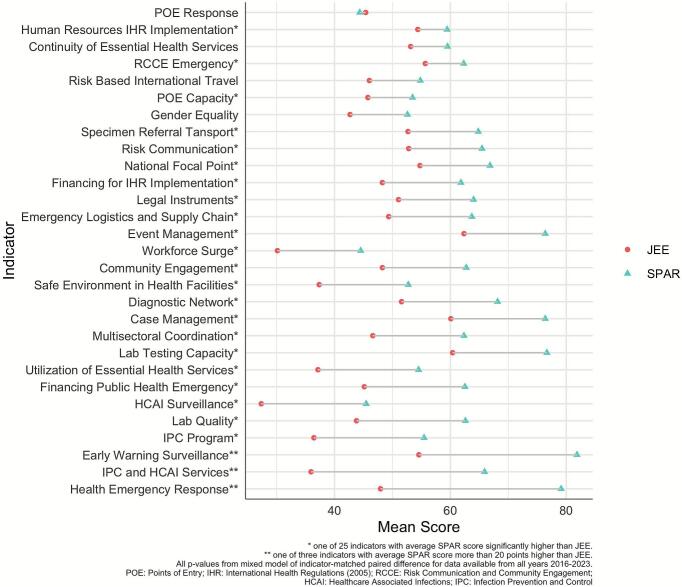
Comparison of 29 matched indicator scores between SPAR and JEE from 2016 to 2023.

Fig. 3 shows the respective scores in SPAR and JEE for the 29 indicators included and matched across the 2016 to 2023 period. Considering all data from 2016 to 2023, four indicators showed agreement between SPAR and JEE scores across all countries (95% CI including zero): risk-based travel, continuity of essential health services, gender equality, and points of entry response. There were 25 indicators that showed SPAR scores were higher than JEE scores on average [Fig. 3, Annex Table 4]. The largest disagreement between scores, with average SPAR score more than 20 points higher than JEE, was seen with early warning surveillance, health emergency response, and infection prevention control (IPC)/healthcare associated infections (HCAI) prevention and control programs. However, these indicators saw improvement in the later editions [Annex Table 5-8]. Across all indicators major improvements were visible in the most recent 2022–2023 edition where 25 of the 28 matched indicators agreed on average between SPAR and JEE scores [Annex Table 5].

There were nine indicators that were consistently defined across all editions and years: early warning surveillance, human resources for IHR implementation, laboratory testing capacity, legal instruments, multisectoral coordination, national focal point, points of entry capacity, points of entry for response, and risk communication for emergencies [Annex Fig. 3]. Seven of these nine indicators showed significant disagreement in the 2016–2018 editions (all except human resources for IHR implementation and points of entry for response) [Annex Table 5]. However, as observed across all indicators, there were major improvements in alignment between SPAR and JEE scores such that by 2022–2023 eight of the nine indicators no longer had a statistically significant difference on average (Annex Table 8).

## 5 Discussion

This study presents a thorough quantitative examination of the alignment between indicator-level scores from SPAR and JEE, two prominent tools for assessing country level public health security preparedness capacity. Our analysis that included 108 paired assessments from 93 States Parties (2016–2023) shows that SPAR scores tend to be higher than JEE scores, but consistency has improved, particularly in the latest 2022–2023 editions. This evolution reflects the ongoing iterative enhancements to these tools, driven by continuous evaluation efforts by States Parties, WHO Secretariat, stakeholders, and technical experts to improve these tools. Our findings on the alignment and refinement of SPAR and JEE are particularly relevant to the IHR amendments adopted in 2024 and the Pandemic Agreement adopted in 2025. [20], [21] The continuous improvement of these assessment tools supports the enhanced transparency and accountability frameworks established by these key global health security instruments.

This analysis included 29 matched indicators between SPAR and JEE, advancing previous analyses of these assessment tools. For example, Razavi et al. reported 13 matched indicators in the 2nd edition of both tools, Tsai et al. analyzed 23 matched indicators between the IHR Monitoring Questionnaire and JEE's 1st edition, Stone et al. identified 3 exact and 11 total capacity alignments with SPAR 1st edition compared to JEE 2nd edition, and Kandel et al. found concordance across 13 capacities between IHR Monitoring Questionnaire and JEE 1st edition in 2017 as well as the SPAR 1st edition and JEE 2nd edition in 2018. [11], [12], [13], [22]. Our research advances previous empirical studies by examining all tool editions, revealing greater alignment in number of matched indicators in recent editions.

Consistent with prior findings, this study indicates that SPAR assessments generally report higher scores than those from JEE, likely a systematic difference attributable to the self-assessment nature of SPAR. This initial, large disparity (20 points in 2016–2017, closely mirroring the findings of Kandel et al., which reported a global mean difference of 18 in 2017), reflects not only the inherent bias of self-reporting, but also fundamental differences in the original objectives of each tool that impact capacity and indicator scope in earlier editions. [13] Subsequent SPAR and JEE editions in 2018–2020 showed a lower mean difference of 8 points, larger than the 0.5 points difference from Kandel et al.'s observation in 2018 likely due to additional data beyond 2018 included in this analysis. Our analysis continued to the latest editions of SPAR and JEE in 2022–2023 to show a gap with SPAR being 6 points higher on average compared to JEE. This closer agreement was likely facilitated by the adoption of an incremental scoring scheme in the most recent editions, a factor highlighted by Linder et al.'s detailed work on the longitudinal mapping of common themes across SPAR editions [23]. This progressive and substantial convergence in both conceptual alignment measured by number matched indicators and score across editions demonstrates the tools' evolution toward supporting their complementary function within the IHRMEF: providing required annual accountability (SPAR) while enabling robust, external core capacity reviews (JEE).

A distinctive component of this study involves analyzing the concordance between SPAR and JEE across regions and income groups. Our results showed improved SPAR-JEE score agreement in recent editions across most regions, except for the European region, where agreement decreased. Regional variation may stem from differing levels of support or guidance in completing the SPAR self-assessment, as well as the voluntary nature and financial support required to undertake a JEE. Notably, our study lacks representation from the Americas region where only two JEEs were completed. This specific finding may arise from the unique application in the Americas region of SPAR as a basis for voluntary external evaluations similar to JEE. [24] Future analyses should investigate whether using SPAR as a basis for voluntary external evaluations could be a successful way to support IHR core capacity assessments and reporting.

This study also examined SPAR and JEE score association by capacity level. Indicators with Level 3 and 4 scores (range 40–80) showed the most disagreement, potentially reflecting the scoring methodology's bounded nature which limits differences at capacity level extremes where few States Parties are realistically below Level 2 or consistently at Level 5. Variability at Levels 3 and 4 may also reflect qualitative attribute descriptions within this mid-range. This divergence creates ambiguity, challenging countries to identify genuine gaps and make appropriate investments. Conversely, the analysis spotlights substantial improvements in agreement for IPC/HCAI indicators following their decoupling in recent editions. This evolution showcases the effective refinement to identify gaps in the assessment tools, ensuring a more precise and tailored evaluation of these critical public health security components. Ultimately, establishing alignment is a necessary foundation for validating both measures and is an essential step needed to address the criticism that preparedness scores did not have a clear association with COVID-19 outcomes [8], [9], [10]. The next steps to continue these improvements are to explore the qualitative data for indicators and States Parties with significant score differences (>20), to understand how to further refine these assessments for global public health security.

Considering the broader objectives, there is no gold standard measurement of public health security capacities. While JEE benefits from external expert evaluation, the outcomes depend on the external team's composition and expertise, who often apply global evidentiary standards and functional capacity requirements in their evaluations. Conversely, SPAR self-assessment reliability relies on the focal point's familiarity with the tool as well as the extent of sub-national and multi-sectoral involvement. These factors likely contribute to the observed score disparities where external teams using global standards may assign lower capacity scores in the case where self-assessors may interpret the presence of a capacity as sufficient. Additionally, slight discrepancies in indicator level definitions between tools can cause score divergence even if the methodology were identical. Since this study analyzed quantitative scores of match indicators, we recommend a future qualitative review of indicator level definitions across tools, prioritizing areas with higher score discrepancies to better align indicator level definitions and concepts.

Despite these factors, Fukuda-Parr notes that IHR required reporting not only addresses global accountability but also facilitates country capacity building. [10] SPAR's self-assessment nature, though potentially less rigorous, enables annual self-reflection, self-learning, and developing policy strategies for strengthening national capacities. Both SPAR and JEE engage professional and organizational networks within the country and across borders, which may in itself strengthen national capacities particularly those with multisectoral dependencies. These reasons help explain the average rise in SPAR scores since their initiation, which also report progress via the SDG indicator 3.d.1. While assessing how well SPAR and JEE measure actual preparedness or resilience in relation to pandemic outcomes is a crucial long-term question, it presents a complex analytical challenge. [25] Such correlations are influenced by numerous other factors in the response pathway, such as data reporting, extending beyond initial capacity assessments. [26] Our study establishes a foundational understanding of capacity assessment tool alignment, which is an essential prerequisite for more complex analyses of the predictive validity of these fundamental IHRMEF tools. Importantly, SPAR annual assessments and JEE voluntary evaluations recommended every 4–5 years are only part of IHRMEF's full spectrum, which also include Simulation Exercises (SimEx) and Action Reviews (Early, Intra, and After) for functional capability assessments. [27], [28] A comprehensive understanding of preparedness requires integrating these diverse evaluations, including human-animal-environment interface assessments such as One Health National Bridging Workshops. Further work on integration of these assessments into a common catalogued score, using Linder et al.'s work of mapping common themes across SPAR editions and Nguni et al.'s methodology of cross-mapping IHRMEF assessments, would enable more coherent and interpretable core capacity evaluations ultimately improving global public health security strengthening efforts where they are most needed. [23], [29]

## 6 Strengths, limitations, and future directions

The main strength of this analysis is its longitudinal design across multiple editions of SPAR and JEE tools in 93 States Parties, enabling insights on trends, improvements, and persisting challenges in global health security preparedness evaluations. However, the study is limited by the evolution of the tools; the initial IHR Monitoring Questionnaire did not use the 1–5 level scoring system later implemented in SPAR and all JEE editions, rendering scores from the years 2016 to 2018 less comparable by design. Moreover, mapping between SPAR and JEE was completed at the indicator level but did not consider variations in definitions at the attribute level. Thus, future research should prioritize qualitative analysis to investigate these attribute-level differences, particularly for indicators with high score discrepancies, to further refine global standards.

Operational factors also limit generalizability. Conducting a JEE is a complex process, as the country must meet a series of steps, including country leadership willingness, dedicated funding, and external team support. These requirements limit the application of JEE and likely contributed to the low participation in the Americas regions (only two JEEs completed) and restricted evaluations during the COVID-19 pandemic (only three JEEs conducted between January 2021 and June 2022), whereas there was nearly universal annual reporting of SPAR (184 States Parties in 2021). This imbalance limits our ability to analyze indicator alignment in all regions and time periods. Fundamentally, all analyses must consider that SPAR and JEE serve distinct purposes, with SPAR being a mandatory reporting mechanism for States Parties to fulfill IHR obligations, while JEE is a voluntary external evaluation conducted every 4–5 years in the perspective of the development of a National Action Plan for Health Security, and this difference could impact their assessment score interpretation.

Despite these limitations, the findings have significant implications for the IHR (2005) and WHO GPW14 objectives. Our analysis reveals high concordance between SPAR and JEE for countries with low capacity indicator scores (<40), providing validated identification of underserved populations truly requiring support. Unlike mid-level capacities where misaligned scores could create ambiguity, the agreement at the lower bounds confirms genuine capacity gaps. As SPAR serves as the data source for SDG 3.d.1 and primary indicator tracking the WHO GPW14 Triple Billions targets, improving its reliability and coherence with complementary tools like the JEE is essential for validating global monitoring and ensuring investments target the populations most in need.

## 7 Conclusion

In conclusion, our analysis confirms significant and progressive improvement in the alignment between SPAR and JEE tools, particularly in recent editions. This study contributes to the evidence base by quantifying how tool refinements have increased the number of matched indicators and reduced score disparity. Further research, incorporating both quantitative and qualitative approaches, is necessary to fully investigate the implications of this improved alignment and to inform the continued improvement of these essential assessment tools. Strengthening the reliability of these tools will aid in accurate interpretation of results, supporting countries in strengthening necessary IHR core capacities for better global health security preparedness.

## CRediT authorship contribution statement

**Kamal Bishowkarma:** Writing – review & editing, Writing – original draft, Visualization, Methodology, Investigation, Formal analysis, Data curation. **Cynthia Bell:** Writing – review & editing, Writing – original draft, Visualization, Supervision, Methodology, Investigation, Formal analysis, Data curation, Conceptualization. **Shanlong Ding:** Writing – review & editing, Data curation. **Robert Nguni:** Writing – review & editing, Methodology, Formal analysis, Data curation. **Luca Vernaccini:** Writing – review & editing, Methodology. **Peter Mala:** Writing – review & editing. **Stéphane de la Rocque:** Writing – review & editing. **Jun Xing:** Writing – review & editing. **Dick Chamla:** Writing – review & editing. **Mary Stephen:** Writing – review & editing. **Reuben Samuel:** Writing – review & editing. **Ihor Perehinets:** Writing – review & editing. **Phuong Nam Nguyen:** Writing – review & editing. **Nirmal Kandel:** Writing – review & editing, Writing – original draft, Supervision, Methodology, Investigation, Conceptualization. **Stella Chungong:** Writing – review & editing, Writing – original draft, Supervision, Investigation.

## Disclaimer

The authors are staff members of the World Health Organization. The authors alone are responsible for the views expressed in this article and they do not necessarily represent the decisions, policy or views of the World Health Organization.

During the preparation of this work the authors used Gemini in order to refine the clarity and coherence of specific sections and to cross-reference the text against reviewer comments for completeness. After using this tool/service, the authors reviewed and edited the content as needed and take full responsibility for the content of the published article.

## Funding

None.

© World Health Organization 2024.

This is an open access article distributed under the terms of the Creative Commons Attribution IGO License (http://creativecommons.org/licenses/by/3.0/igo/legalcode), which permits unrestricted use, distribution, and reproduction in any medium, provided the original work is properly cited. In any reproduction of this article there should not be any suggestion that WHO or this article endorse any specific organization or products. The use of the WHO logo is not permitted. This notice should be preserved along with the article's original URL.

## Declaration of competing interest

The authors declare that they have no known competing financial interests or personal relationships that could have appeared to influence the work reported in this paper.

## Appendix A Supplementary data

Supplementary material Image 1
