# Supplementary material for: Estimating global public health security preparedness capacity: The contribution of SPAR and JEE
Source: Dialogues Health. 2026 Jun;8:100282. doi: 10.1016/j.dialog.2026.100282 (PMC12865538; doi:10.1016/j.dialog.2026.100282)
Supplement: Supplementary file 1 — Supplementary material [file mmc1.docx]

Supplementary Material

Annex Table 1: SPAR indicator mapping across editions

Annex Table 2: JEE indicator mapping across editions

Annex Table 3: Mapping of indicators between SPAR version 2 and JEE version 3

Annex Table 4. All 29 matched indicators between SPAR and JEE across 2016-2023

| Indicator | Mean Difference  [95% CI], n | Average JEE and SPAR |
| --- | --- | --- |
| Global | 13.2 [10.3, 16.0] | 56.3 |
| health_emergency_response | 30.9 [25.4, 36.3], n=77 | 64.9 |
| ipc_hcai_services | 30.1 [23.8, 36.4], n=52 | 53.5 |
| early_warning_surveillance | 26.9 [22.3, 31.6], n=108 | 67.4 |
| ipc_program | 17.8 [8.4, 27.1], n=22 | 40.9 |
| lab_quality | 17.1 [6.8, 27.4], n=22 | 49.5 |
| hcai_surveillance | 16.8 [7.5, 26.2], n=22 | 31.4 |
| financing_ph_emergency | 16 [6.2, 25.8], n=22 | 49.5 |
| health_services_use | 16 [6.2, 25.8], n=22 | 42.4 |
| lab_testing_capacity | 15.9 [11.2, 20.6], n=108 | 68.2 |
| multisectoral_coordination | 15.4 [10.7, 20], n=108 | 53.7 |
| case_management | 15 [5.6, 24.4], n=22 | 63.2 |
| diagnostic_network | 14.9 [4.6, 25.1], n=22 | 55.0 |
| safe_env_hf | 14.1 [4.7, 23.5], n=22 | 40.0 |
| community_engagement | 13.2 [3.8, 22.6], n=22 | 50.5 |
| workforce_surge | 13 [3.2, 22.8], n=22 | 32.9 |
| event_management | 12.8 [3.4, 22.1], n=22 | 64.3 |
| logistic_emergency | 12.7 [2.4, 22.9], n=22 | 54.0 |
| legal_instruments | 12.5 [7.8, 17.3], n=108 | 56.9 |
| ihr_imp_financing | 12.3 [2.9, 21.7], n=22 | 50.0 |
| national_focal_point | 11.7 [7.1, 16.4], n=108 | 60.0 |
| risk_communication | 11.4 [2, 20.8], n=22 | 54.1 |
| specimen_referral_tansport | 10.4 [0.2, 20.7], n=22 | 56.0 |
| gender_equality | 8.2 [-2, 18.5], n=22 | 44.5 |
| poe_capacity | 7.3 [2.6, 12], n=108 | 49.3 |
| risk_based_int_travel | 7.1 [-3.2, 17.4], n=22 | 47.5 |
| rcce_emergency | 6.3 [1.6, 10.9], n=108 | 58.2 |
| continuity_ehs | 5 [-4.8, 14.8], n=22 | 51.9 |
| hr_ihr_implementation | 4.7 [0, 9.4], n=108 | 56.1 |
| poe_response | -1.4 [-6.1, 3.3], n=108 | 44.6 |

Annex Table 5: Eleven matched indicators between SPAR and JEE for 2016 to 2017 period

| Indicator | Mean Difference [95% CI], n = 52 | Average JEE and SPAR |
| --- | --- | --- |
| Global | 19.6 [16.0, 23.1] | 60.5 |
| health_emergency_response | 41.1 [34.7, 47.5] | 62.9 |
| early_warning_surveillance | 38.8 [32.4, 45.2] | 71.7 |
| ipc_hcai_services | 33.9 [27.6, 40.3] | 53.5 |
| lab_testing_capacity | 25.5 [19.1, 31.9] | 73.1 |
| multisectoral_coordination | 22.9 [16.6, 29.3] | 56.5 |
| national_focal_point | 15.6 [9.2, 21.9] | 66.8 |
| legal_instruments | 15.6 [9.2, 21.9] | 62.4 |
| poe_capacity | 12.6 [6.2, 18.9] | 51.3 |
| rcce_emergency | 7.1 [0.7, 13.4] | 64.3 |
| hr_ihr_implementation | 1.9 [-4.5, 8.3] | 59.8 |
| poe_response | 0.1 [-6.3, 6.5] | 43.5 |

Annex Table 6: Nine matched indicators between SPAR and JEE for 2018 to 2020 period

| Indicator | Mean Difference [95% CI], n = 31 | Average JEE and SPAR |
| --- | --- | --- |
| Global | 8.5 [3.7, 13.3] | 53.3 |
| early_warning_surveillance | 21.0 [12.7, 29.2] | 60.0 |
| multisectoral_coordination | 13.2 [4.9, 21.5] | 49.0 |
| legal_instruments | 11.6 [3.3, 19.9] | 53.7 |
| hr_ihr_implementation | 8.7 [0.4, 17] | 53.9 |
| national_focal_point | 7.7 [-0.5, 16] | 58.2 |
| lab_testing_capacity | 6.8 [-1.5, 15] | 65.2 |
| poe_capacity | 4.8 [-3.4, 13.1] | 43.5 |
| rcce_emergency | 3.5 [-4.7, 11.8] | 54.5 |
| poe_response | -1.0 [-9.2, 7.3] | 41.9 |

Annex Table 7: Ten matched indicators between SPAR and JEE for 2021 to 2022 period

| Indicator | Mean Difference [95% CI], n = 3 | Average JEE and SPAR |
| --- | --- | --- |
| Global | 10.7 [-4.5, 25.8] | 63.3 |
| health_emergency_response | 40 [13.4, 66.6] | 66.7 |
| early_warning_surveillance | 26.7 [0.1, 53.2] | 66.7 |
| lab_testing_capacity | 20 [-6.6, 46.6] | 70.0 |
| rcce_emergency | 13.3 [-13.2, 39.9] | 53.3 |
| hr_ihr_implementation | 13.3 [-13.2, 39.9] | 66.7 |
| legal_instruments | 6.7 [-19.9, 33.2] | 63.3 |
| multisectoral_coordination | 6.7 [-19.9, 33.2] | 63.3 |
| national_focal_point | -6.7 [-33.2, 19.9] | 70.0 |
| poe_response | -6.7 [-33.2, 19.9] | 56.7 |
| poe_capacity | -6.7 [-33.2, 19.9] | 56.7 |

Annex Table 8: Twenty-eight matched indicators between SPAR and JEE for 2022 to 2023 period

| Indicator | Mean Difference [95% CI], n = 22 | Average JEE and SPAR |
| --- | --- | --- |
| Global | 5.6 [0.6, 10.6] | 49.7 |
| ipc_program | 11.3 [1.5, 21] | 40.9 |
| hcai_surveillance | 10.3 [0.6, 20.1] | 31.4 |
| national_focal_point | 10.3 [0.6, 20.1] | 45.0 |
| lab_quality | 10.3 [-0.3, 20.9] | 49.5 |
| financing_ph_emergency | 9.3 [-0.8, 19.4] | 49.5 |
| health_services_use | 9.3 [-0.8, 19.4] | 42.4 |
| case_management | 8.5 [-1.2, 18.3] | 63.2 |
| diagnostic_network | 8.1 [-2.5, 18.6] | 55.0 |
| safe_env_hf | 7.6 [-2.1, 17.4] | 40.0 |
| legal_instruments | 6.9 [-3.6, 17.5] | 46.5 |
| rcce_emergency | 6.7 [-3, 16.5] | 49.5 |
| early_warning_surveillance | 6.7 [-3, 16.5] | 67.7 |
| community_engagement | 6.7 [-3, 16.5] | 50.5 |
| workforce_surge | 6.3 [-3.8, 16.4] | 32.9 |
| event_management | 6.3 [-3.5, 16] | 64.3 |
| logistic_emergency | 5.8 [-4.7, 16.4] | 54.0 |
| ihr_imp_financing | 5.8 [-3.9, 15.5] | 50.0 |
| risk_communication | 4.9 [-4.8, 14.6] | 54.1 |
| hr_ihr_implementation | 4 [-5.8, 13.7] | 49.1 |
| specimen_referral_tansport | 3.6 [-7, 14.2] | 56.0 |
| health_emergency_response | 3.6 [-7, 14.2] | 70.0 |
| lab_testing_capacity | 3.6 [-7, 14.2] | 59.5 |
| gender_equality | 1.4 [-9.2, 12] | 44.5 |
| multisectoral_coordination | 1.3 [-8.5, 11] | 52.3 |
| risk_based_int_travel | 0.3 [-10.3, 10.9] | 47.5 |
| poe_capacity | -0.8 [-11.4, 9.8] | 52.0 |
| continuity_ehs | -1.7 [-11.8, 8.4] | 51.9 |
| poe_response | -5.3 [-15.9, 5.3] | 49.5 |
| poe_response | -8.9 [-18.6, 0.8] | 49.5 |

Annex Figure 1: Indicator-matched mean difference between SPAR and JEE from 2016 to 2023, by indicators and years/editions


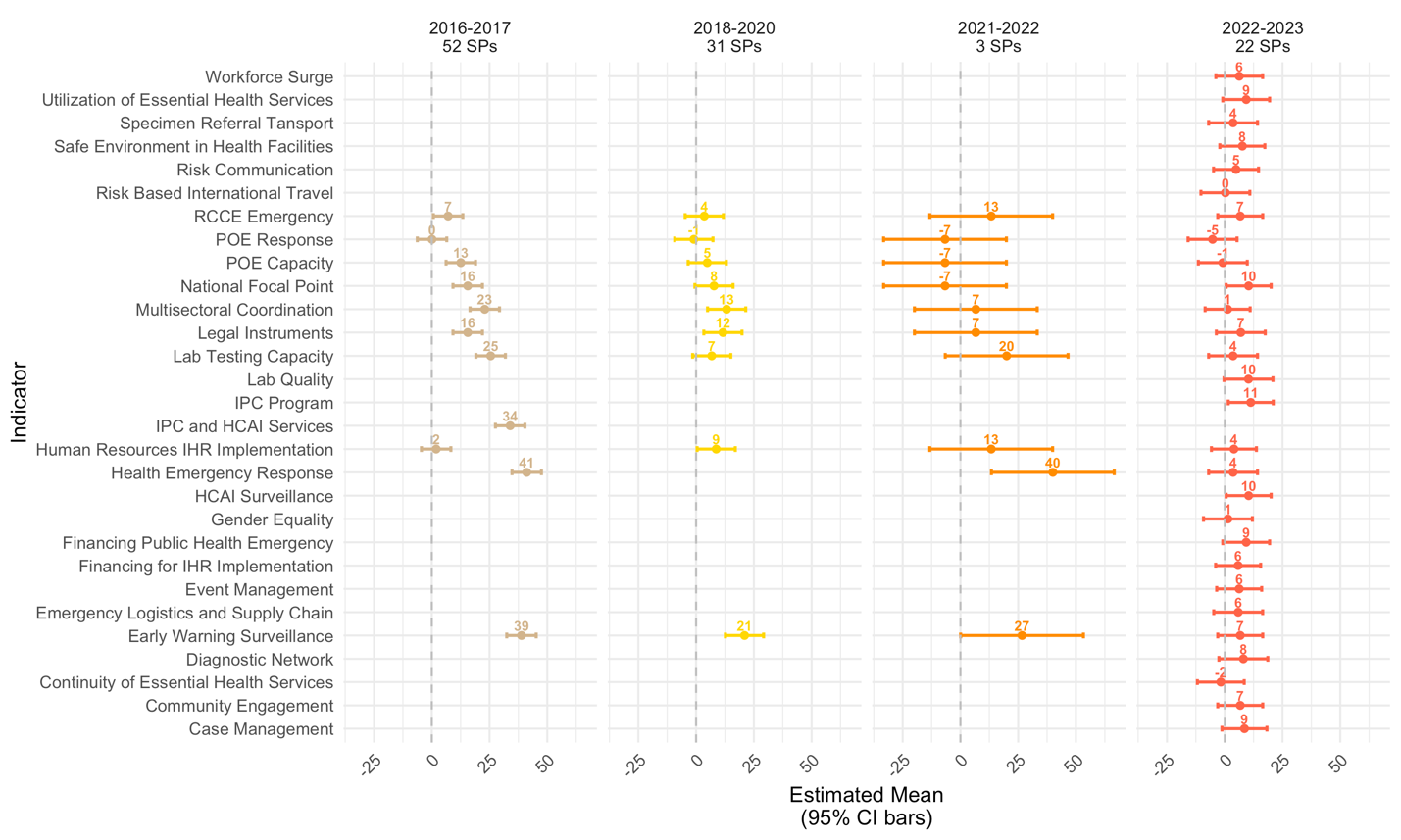


Annex Figure 2: Indicator-matched mean difference between SPAR and JEE from 2016 to 2023, by regions and years/editions
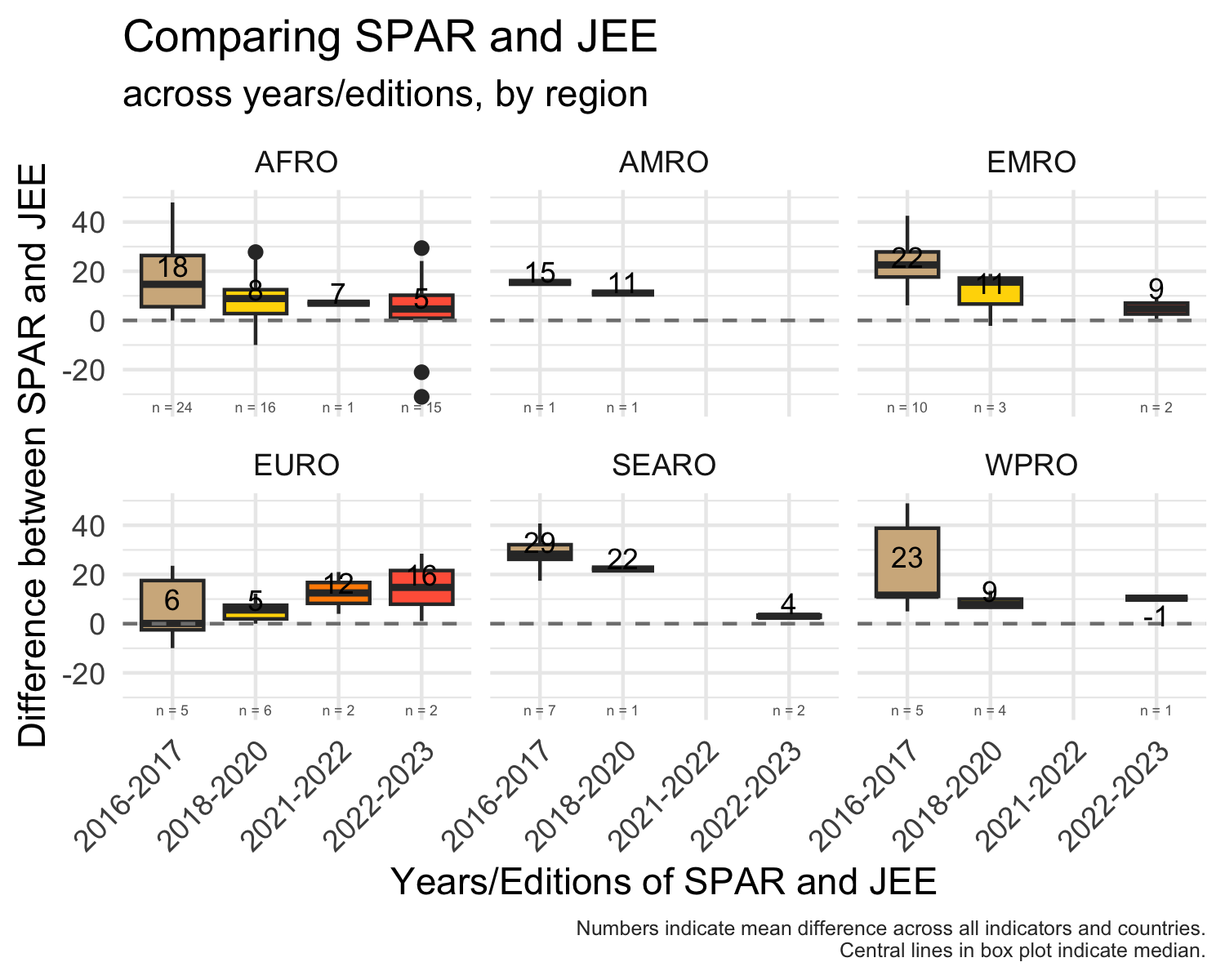


Annex Figure 3: Indicator-matched mean difference between SPAR and JEE from 2016 to 2023, by years/editions

*
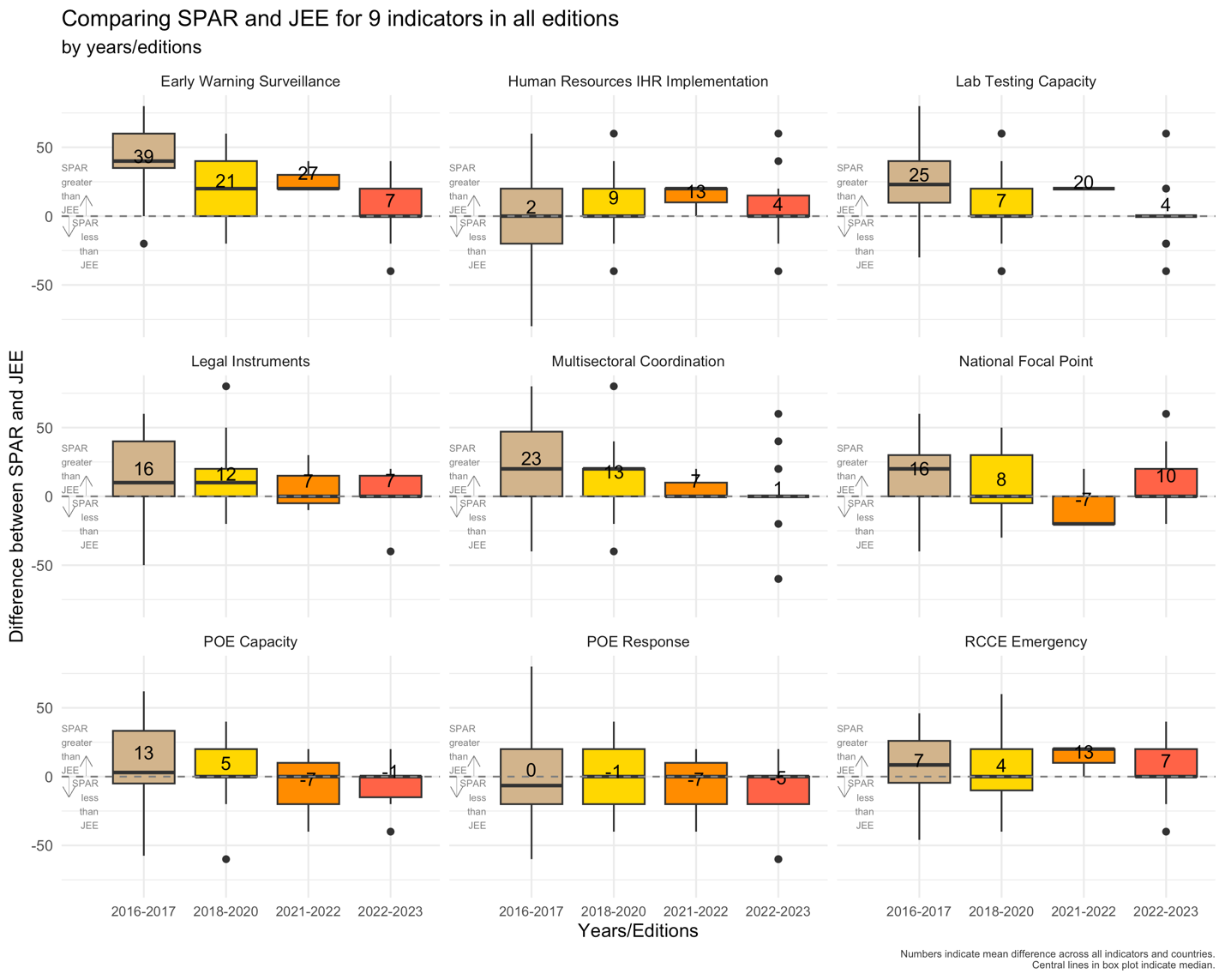
*
